# Efficient single-pixel multispectral imaging via non-mechanical spatio-spectral modulation

**DOI:** 10.1038/srep41435

**Published:** 2017-01-27

**Authors:** Ziwei Li, Jinli Suo, Xuemei Hu, Chao Deng, Jingtao Fan, Qionghai Dai

**Affiliations:** 1 Department of Automation, Tsinghua University, Beijing 100084, China; 2 High-Tech Institute of Xi’an, Xi’an 710025, China

## Abstract

Combining spectral imaging with compressive sensing (CS) enables efficient data acquisition by fully utilizing the intrinsic redundancies in natural images. Current compressive multispectral imagers, which are mostly based on array sensors (e.g, CCD or CMOS), suffer from limited spectral range and relatively low photon efficiency. To address these issues, this paper reports a multispectral imaging scheme with a single-pixel detector. Inspired by the spatial resolution redundancy of current spatial light modulators (SLMs) relative to the target reconstruction, we design an all-optical spectral splitting device to spatially split the light emitted from the object into several counterparts with different spectrums. Separated spectral channels are spatially modulated simultaneously with individual codes by an SLM. This no-moving-part modulation ensures a stable and fast system, and the spatial multiplexing ensures an efficient acquisition. A proof-of-concept setup is built and validated for 8-channel multispectral imaging within 420~720 nm wavelength range on both macro and micro objects, showing a potential for efficient multispectral imager in macroscopic and biomedical applications.

Spectral imaging aims to record a scene/sample with a 3D data cube (*x, y, λ*), describing its intensity of each location (*x, y*) at each wavelength *λ*^1^. With the ability to resolve both spatial and spectral information, multispectral imaging has significant potential for applications such as material identification^2^ and biological observation^3^.

Naturally, multispectral imaging techniques suffer from compromise among spatial, spectral and temporal resolutions. Conventional multispectral imagers usually segregate either spatial or spectral information and measure via temporal scanning^4^. The full measurement of spatio-spectral data in such a scanning manner requires long acquisition time and huge amount of data to be stored and transfered. However, these strategies neglect the low intrinsic dimension of the data cube along both spatial and spectral dimensions^5^, and the redundancy motivates researchers to conduct multispectral imaging in a more data efficient manner. Compressive sensing (CS) is an efficient signal acquisition scheme that enables high quality reconstruction of the redundant signals under far lower sampling rates than required by the Nyquist-sampling Theorem^6^,^7^. Considering the redundancy of the 3D spatio-spectral data cube, it is reasonable to apply CS technique to spectral imaging to reduce the acquisition time.

Implementations of CS-based spectral imaging have been proposed recently, following either spatio-resolved sensor (for example, using a CCD/CMOS for detecting)^8^,^9^,^10^,^11^,^12^ or single-pixel photodiode structure^13^,^14^,^15^,^16^,^17^. In this paper, we focus on single-pixel CS due to the following advantages: (1) the photon efficiency of a photodiode is higher than that of the array sensors since spatio-spectral information is multiplexed and collected by a single detection unit^7^,^18^; (2) single-pixel detector is available for sensing at regions of the electromagnetic spectrum, such as infrared^19^ or terahertz^20^, whereas applications for spatio-resolved architectures require expensive cameras. Among studies on single-pixel spectral imaging, one approach directly extend the grayscale single-pixel imaging architecture by additionally resolving spectral information at the detector module. These approaches either require a chromatic detector such as a cormercially available spectrometer^13^,^14^ or sweeping different spectrum bands with spectral filters^15^. The former is expensive and bulky, and the latter is of low photon efficiency and inflexible for large number of spectral bands. Another type of implementations collect encoded spatio-spectral signals in a multiplexed manner. Wang *et al*. temporally multiplex spectra into the duration time of each illumination pattern using a six-segment color wheel^16^. Bian *et al*. proposed a rolling disk scheme that multiplexes frequency modulated spectral channels into one measurement and demultiplexes them in the Fourier domain^17^. Although these strategies enable faster and more photon-efficient acquisitions, the use of mechanical modulation leads to unstable and complex implementations, especially in high speed operation.

Here, we propose a multispectral imaging scheme under a single-pixel CS architechture with an all-optical multiplexing technique instead of mechanical modulation. Single-pixel CS systems use a spatial light modulator (SLM) to modulate spatio-resolved light into structured pattern, whose resolution is the same as that of the reconstructed image. Inspired by the spatial resolution gap between current SLMs (e.g., 1920 × 1080 pixels for Texas Instruments device: DLPNIRSCANEVM,.9XGA DMD) and target reconstructions (whose dimension are mostly in the order of 100 × 100 pixels), we integrate CS technique of different spectral bands in a spatial multiplexing manner. Specifically, the light emitted from the scene is first divided and spatially mapped to different subregions after travelling through an optical splitter introduced by refs 21 and 22, with each counterpart of distinguishing spectral distribution. Separated channels then enter an SLM and get coded simultaneously by independent patterns. The summation of spectral information for all channels is collected as one measurement by a single-pixel detector, and this ensures a high signal-to-noise ratio (SNR) compared to systems that record spectral information onto separate detectors or sequentially onto a single detector. To fully utilize the sparsity both in spatial domain and along spectral direction, 3D compressive sensing algorithm is adopted to reconstruct the multispectral information.

## Results

### Schematic and acquisition

**Figure 1:**
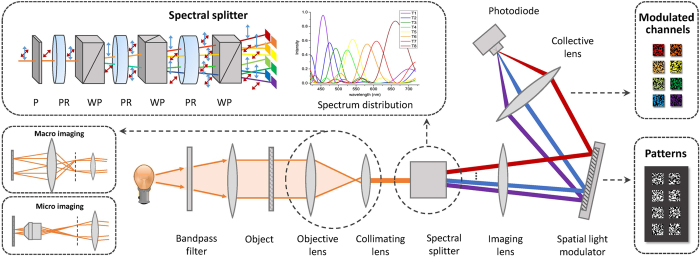
Schematic setup of proposed multispectral imaging system. The light first illuminates the object and propagates through an all-optical spectral splitter, which separates light into eight parts with different spectral distributions. The splitter is composed of a polarizer (P) and three alternatively placed Wollaston prisms (WP) and phase retarders (PR). The 1920 × 1080-pixel SLM encodes each channel with independent patterns and a single-pixel detector records multiplexed multi-channel information simultaneously. The system is applicable to both macroscopic scenes and microscopic specimens, differentiating in objective lens.

The optical configuration of our system is schematically illustrated in Fig. 1. The configuration is essentially a single-pixel system operating on multiple channels simultaneously. Spatially and spectrally distinguished signals are individually modulated on the SLM plane, and the coded multispectral information is effectively recorded without sequential scanning.

Illumination light generated by a broadband white light source is first filtered to the wavelength range of 420~720 nm by a band-pass filter, and then gets converged by a collective lens to illuminate the target object. Emitted light coming from the object plane then propagates through an objective lens (convex lens for macro objects or microscopic objective for micro samples), gets colliminated at a proper beam size, and reaches the spectral splitting device. Instead of mechanical translation or filtering device, we spatially split the multispectral components by an optical splitter. The splitter is assembled with a polarizer and a cascade of alternatively placed phase retarders and Wollaston polarizing beam splitters, as shown in the topleft inset of Fig. 1. The polarizer first converts colliminated light into linearly polarized light. The following phase retarder changes the polarization state of outgoing light into a function of the wavelength. Considering a phase retarder with thickness *d*, and the ordinary and extraordinary refractive indexes *n*_*o*_ and *n*_*e*_, the phase shift for wavelength *λ* is given by Δ = *π*|*n*_*o*_ − *n*_*e*_|*d*/*λ*. When propagates through a Wollaston prism, light is splitted into two orthogonal linearly polarized beams (noted as parallel and orthogonal polarizations for simplicity), and separated in propagation directions by a small separation angle *θ*. The spectral transmission functions of the parallelly and orthogonally polarized beams through a phase retarder and Wollaston prism pair can be respectively expressed as


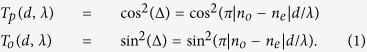


Adjacent Wollaston prisms are placed with their splitting directions perpendicular to each other, and this layout splits light along vertical and horizontal directions alternately. For a three level retarder-prism pairs employed in our work, eight channels are splitted in 2 × 4 layout with distinguishing spectral distributions as


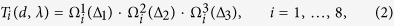


where Δ_*k*_ = *π*|*n*_*o*_ − *n*_*e*_|*d*_*k*_/*λ, k* = 1, 2, 3 and 
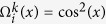
 or sin^2^(*x*) depending on the polarization state of *i*th channel when it propagates through the *k*th prism. The thicknesses for the quartz phase retarders are optimally chosen in our experiments to produce eight spectral bandpasses approximately equally spaced within the wavelength range 420~720 nm. Corresponding spectral response curves are plotted in Fig. 1, with the main lobes for all channels distributing nearly uniformly. Minimizing overlap among main lobes ensures reliable demultiplexing.

Separated channels then arrive at a tube lens and get imaged on the SLM. Instead of temporal multiplexing which requires long acquisition time, we conduct spatial multiplexing by modulating different channels with different patterns. Figure 1 shows an exemplar binary pattern displayed on the SLM. Eight sub-patterns are placed correspondingly in 2 × 4 layout, with the positions determined by the locations and separation angles of the Wollaston prisms. For each round of acquisition, the eight sub-patterns are updated simultaneously. To meet the incoherent sampling condition raised by CS theory^6^, sub-patterns for different channels are required to be independent of each other. Binary Gaussian random codes are utilized in our work for its high incoherency.

### Multispectral imaging results

To demonstrate the effectiveness of the proposed system, we conduct experiments on a number of colored objects. The resolution of the target objects is fixed to 128 × 128 pixels, and a 1920 × 1080-resolution fast SLM (Texas Instrument DLP Discovery 4100,.95XGA DMD) is used to spatially modulate eight channels. The SLM works at 10 kHz with 25% blank time (displaying an all-zero pattern) for synchronization, and the sampling rate of the single-pixel detector (Thorlabs PDA100A-EC Silicon photodiode, 340~1100 nm) is set to be 50 MHz. A self-synchronized sampling scheme^23^ is utilized to synchronize the SLM and the detector. For each object, 4915 high-resolution patterns (equivalently, 39320 low-resolution Gaussian random binary patterns for 8 channels) are used for multispectral image reconstruction, and the corresponding compression ratio is 0.3 comparing to the full measurement of the spectral cube (*N* = 128 × 128 × 8). It takes around 0.5 s for the data acquisition and the reconstruction is conduct offline. The working spectral range is set to be 420~720 nm by filtering the light from the white light source (Energetiq LDLS EQ-1500) with a bandpass filter.

**Figure 2:**
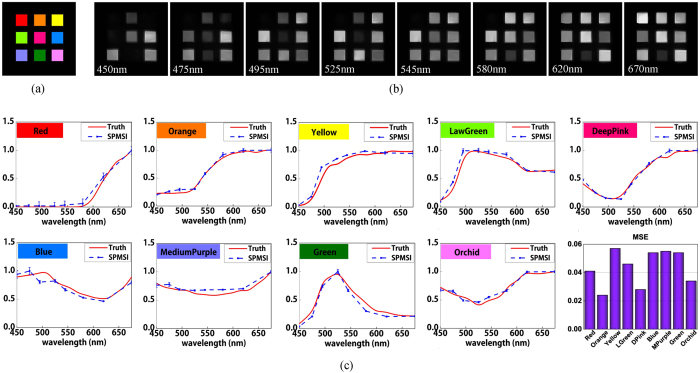
Reconstructed multispectral images of color checker film. (**a**) is the ground truth color film. (**b**) shows eight spectral channels. (**c**) shows spectrum comparisons between the reconstructed results of our single-pixel multispectral imager (SPMSI) and the ground truth measured by a spectrometer. Red solid curves represent the ground truth and blue symboled dashed curves represent reconstructed spectrums.

The first experiment is conducted on a printed color checker film with nine patches (see Fig. 2(a)). The size of the film is around 20 × 20 mm^2^ and each single channel imaged on the SLM is around 2.7 × 2.7 mm^2^. To fit the size of the images, 256 × 256 pixels on the SLM are used to display a 128 × 128 sub-pattern with 4 neighboring pixels binned as one. We also calibrare the spherical aberration of the imaging lens and correct the distortion of the bordering channels. Eight multispectral images discretized in the range 420~720 nm are succesfully reconstructed from the multiplexed measurements, as presented in Fig. 2(b). Nine color patches are recovered with high quality. The promising results demonstrate the effectiveness of the proposed multispectral imaging approach.

To further evaluate the accuracy of our approach, we conduct quantitative analysis on the reconstructed images. Each patch on the target film is monochrome, thus we take the average of all pixels within each color patch as the reconstructed spectrum for the certain color. Figure 2(c) shows the reconstructed spectral response curves linearly interpolated on eight discrete wavelengths for all nine tested colors. Ground truth spectrums are measured by a compact spectrometer (Thorlabs CCS200/M, 200~1000 nm) and presented at an interval of 10 nm. The spectral responses are both normalized to the range [0, 1] and the standard deviation of each color patch is calculated and illustrated with error bars. As shown, our reconstructed spectrums are of quite close intensity distributions with the ground truth. The root mean square errors (RMSE) between reconstructed spectrums and ground truth are 0.041, 0.024, 0.057, 0.046, 0.028, 0.054, 0.055, 0.054 and 0.034 for nine color patches, respectively, as illustrated in bottomright inset of Fig. 2(c). Small deviations from the ground truth demonstrate that our system is of high spectral accuracy.

**Figure 3:**
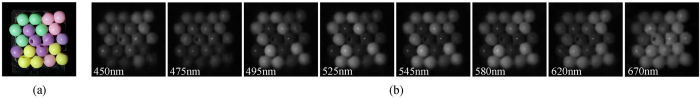
Reconstructed multispectral images of multi-color beads. (**a**) is the ground truth color image captured by CCD camera under sunlight. (**b**) shows eight reconstructed spectral channels.

In the following experiment, we illuminate a approximately vertically placed 20 × 20 mm^2^ scene which includes a layer of multi-color beads (seen in Fig. 3(a)) with the spectrally filtered light source, and collect reflective light for spectral splitting, mapping and modulation. Reconstructed multispectral images are presented in Fig. 3(b). Since the beams are placed slightly tilted from the vertical imaging plane, defocusing appears at the top and bottom edges. It is clear that the responses of green beads are higher in the range of 495~545 nm, while the responses of pink beads are higher in the range of 450~475 nm and 580~670 nm. The high quality reconstruction demonstrates our ability to achieve multispectral imaging on real scenes.

**Figure 4:**
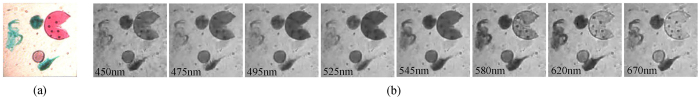
Reconstructed multispectral images of Volvox specimen. (**a**) is the ground truth color image captured by CCD camera. (**b**) shows eight reconstructed spectral channels.

To validate the effectiveness of our approach in microscopy, we further conduct experiments on a multicolor dyed Volvox specimen. To visualize the micro-scale structures of the specimen, a microscope objective lens (10 × 0.25 NA) is utilized in place of the convex lens in above experiments. The successive achromatic lens (L = 30 mm) colliminates the light from the image at the back focal plane of the objective. Multi-channel images are then focused to the SLM by a tube lens. We captured a reference color image by an RGB CCD camera (PointGrey FL2-08s2c), as illustrated in Fig. 4(a), and the eight 128 × 128-pixel spectral images reconstructed by our approach are presented in Fig. 4(b). It is evident that the grand red Volvox colony appears dimmer at low frequency spectrum and brighter at high frequency spectrum, while the green structures are dimmer at medium frequency spectrum and brighter at high and low frequency spectrums. The results validate the applicability of our approach for microscopic imaging.

## Discussion

This paper proposes an efficient multispectral imaging scheme with a single-pixel detector. Utilizing the spatial resolution redundancy of current SLMs, we extend conventional 2D spatial reconstruction to 3D spatio-spectral reconstruction via spatial multiplexing of multiple channels, without introducing additional acquisition time. For the spatial multiplexing, spatial splitting and spectral separation are achieved in an all-optical manner, which avoids the instability and limited response speed of commonly used mechanical modulation techniques. In addition, the single-pixel detector collects the coded accumulation of spectral bands, so the imaging is of high photon efficiency. Experiments validate the ability of our system to resolve spatial and spectral information effectively and accurately. The system holds great potential for developing stable, compact and photon efficient multispectral cameras in the future.

Although we acquire eight channels within the 420~720 nm range in the presented experiments, the system is of high flexibility for extention to 16 or 32 channels by adding another one or two levels of phase retarder-prism pair. It should be mentioned that as the spectral channel number increases, larger Wollaston prisms (e.g, 30 × 30 mm^2^-size) are required. Moreover, the spectral range of our system is adjustable by simply changing the thicknesses of the phase retarders. This shows enormous promise for macroscopy and microscopy imaging within non-visible spectral ranges, under which conventional array sensors may be unavailable or of high cost.

The current prototype is designed to split spectral components with an average bandwidth of 25 nm. Nevertheless, the technique can be utilized for finer spectral resolution imaging by adjusting the composition of the spectral splitter. Experimental results have been demonstrated for retinal imaging with spectral resolution of 5 ± 2 nm within 570~620 nm using the same Lyot filter^21^. Spatial resolution depends on two factors: the NA of the system and the pixel resolution of modulation. In our system setting, the number of modulation pixels for each channel is the dominant limit for spatial resolution. With the effective modulation pixels fixed to be 128 × 128, we can calculate the spatial resolution by dividing the FOV with the pixel number, resulting in 0.16 *mm* for macroscopic imaging and approximately 10 *μm* for microscopic imaging. Higher spatial resolution can be achieved by more modulation pixels, however at the expense of increased acquisition time and computational cost.

Different from other spectral imagers that conduct spatial multiplexing with dispersive devices such as gratings^24^ and prisms^25^ which separate light along one dimension, our system enables a two-dimensional dispersion. This ensures a better use of the SLM’s spatial resolution redundancy. Besides, the single-pixel architecture may produce a more compact camera design. Compared with single-pixel spectral imaging schemes recently proposed by Bian *et al*.^17^ and Wang *et al*.^16^, an important advantage of our system is the elimination of mechanical modulation. These approaches utilized a rolling disk for frequency modulation or spectral separation, which could largly limit the working speed and introduce mechanical vibrations. For example, the frequency multiplexing scheme by Bian *et al*. takes around 1 minute to acquire a 64 × 64 × 10 datacube. As a contrast, our technique can achieve good performance at 2 Hz acquisition for the 128 × 128 × 8 data. Considering the total transmission efficiency, both reported works are limited to no more than 40%, with dominant light losses caused by the grating and frequency modulation for Bian *et al*.‘s approach and the color filters for Wang *et al*.‘s. However, our system reaches a total efficiency up to 50% with half of the light excluded by the polarizer.

So far, the proposed single-pixel spectral system is not fast enough for dynamic scenes, even though spatial and intra-spectral redundancy is utilized to reduce required measurements. We can further accelerate the speed by using advanced pattern designs and content-adaptive schemes^26^. Besides, current spatial resolution remains insufficient for large-scale scenes (especially in biological microscopy) due to the trade-off between spatial resolution and computation cost of CS reconstruction. Fortunately, recent studies have reported on less computation consuming techniques for high resolution reconstruction of single-pixel imaging architectures, such as the phase-shifting sinusoid structured illumination scheme^27^ and the sub-pixel displacement microscanning approach^28^. These strategies provide a possibility for improving the spatial resolution of our system.

## Methods

### Spectral splitter

**Figure 5:**
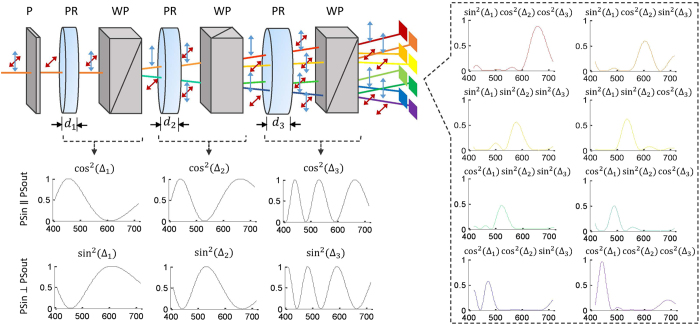
Illustration of the spatial and spectral splitting process for a broadband light beam. The bottomleft shows the associated spectral transmission functions for each pair pf phase retarder and prism, and the right inset shows the spectral response for each bandpass channel. P, polarizer; PR, phase retarder; WP, Wollaston prism; PSin, input polarization state; PSout, output polarization state.

The mapping of 3D spatio-spectral data to spatially splitted 2D spectral channels is achieved by an all-optical splitting device consisting of successive pairs of alternatively placed birefringent phase retarders and Wollaston polarizers. Input linearly polarized broadband light is transformed into nonlinearly polarized by the phase retarder, with the polarization state defined as a function of the wavelength. The nonlinearly polarized beam is then splitted into two orthogonal linearly polarized beams by the polarized prism, differentiating in splitting intensity ratio according to the polarization state. Hence we can conclude that the splitting ratio is wavelength-dependent and the phase retarder-prism pair achieves simultaneous spatial splitting as well as spectral splitting. A schematic drawing illustrating the polarization states for the light before and after passing through each optical element is shown in Fig. 5. In our experiments, calcite Wollaston polarized prisms (20 × 20 mm^2^ in size) with the separation angle *θ* = 10° and quartz phase retarders with the thicknesses *d* = 0.10, 0.145, 0.29 mm are used to assemble the spectral splitter. Spectral transmission functions of each retarder-prism pair result in the forms of cos^2^(·) and sin^2^(·) depending on whether the output polarization state is parallel with or orthogonal to the input (see Eq. (1)), as illustrated in the bottomleft inset of Fig. 5. We can then deduce the transmission functions for eight output beams from the splitter to be the cumulative products of three levels of cos^2^(·) or sin^2^(·) functions, as presented in the right inset of Fig. 5. For each channel, the spectral distribution concentrates in one narrow band, and the main lobes for all channels distribute nearly uniformly in the spectral range, with center frequencies locating at 450, 475, 495, 525, 545, 580, 620 and 670 nm respectively. The slightly nonuniform distribution is inevitable due to the nonlinear relationship between transmissive intensity and wavelength *λ*.

### Multispectral reconstruction

The reconstruction of spatial multiplexed multispectral data is conducted on a three dimensiton (3D) compressive sensing scheme to achieve high reconstruction efficiency. Denoting the sub-patterns for the *m*th measurement as **p**_*m*,1_, **p**_*m*,2_, …, **p**_*m*,8_, the total intensity value of eight modulated channels recorded by the single-pixel detector can be writen as


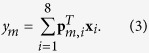


Let *N* = *N*_*x*_ × *N*_*y*_, 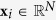 represent the vectorized spectral image of the *i*th channel, *N*_*x*_, *N*_*y*_ denote the resolutions along x and y axis, and *T* represents the transpose operator, after *M* samplings, we record a measurement vector


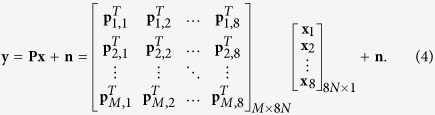


In this equation, 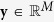, 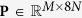 and 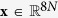 represent the vectorized measurement, sensing matrix and multispectral images, respectively. Here, 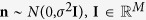 denotes the detection noise vector with i.i.d. entries. To estimate the multiple channels {**x**_*i*_}, we exploit a 3D reconstruction model which imposes the total variation (TV) regularization in both spatial and spectral domains as follows


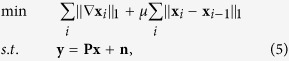


where ∇ represents a two-dimensional TV operator in spatial domain and *μ* is a weighting parameter to balance the TV regulation terms along spatial and spectral dimensions. We fix *μ* to be 0.5 in the following experiments. Equation (5) is a standard convex optimization problem and can be mathmatically solved by multiple algorithms. In our experiments, we adopt Augmented Lagrangian Multiplier algorithm due to its high efficiency for noisy data input^29^ and write the code in Matlab. To reconstruct a 128 × 128 × 8 datacube, approximately 30 seconds is required on a computer with Intel i7-5960X CPU @ 3.0 GHz and 32 GB RAM. The reconstruction process can be further accelerated by using GPUs.

## Additional Information

**How to cite this article**: Li, Z. *et al*. Effcient single-pixel multispectral imaging via non-mechanical spatio-spectral modulation. *Sci. Rep.* **7**, 41435; doi: 10.1038/srep41435 (2017).

**Publisher's note:** Springer Nature remains neutral with regard to jurisdictional claims in published maps and institutional affiliations.

## References

[b1] Garini Y., Young I. T. & Mcnamara G. Spectral imaging: principles and applications. Cytometry Part A 69A, 735–747 (2006). 10.1002/cyto.a.20311 16969819

[b2] Shaw G. A. & Burke H. K. Spectral imaging for remote sensing. Lincoln Laboratory J. 14, 3–28 (2003).

[b3] Zimmermann T., Rietdorf J. & Pepperkok R. Spectral imaging and its applications in live cell microscopy. FEBS Lett. 546, 87–92 (2003). 12829241 10.1016/s0014-5793(03)00521-0

[b4] Bao J. & Bawendi M. G. A colloidal quantum dot spectrometer. Nature 523, 67–70 (2015). 26135449 10.1038/nature14576

[b5] Keshava N. & Mustard J. F. Spectral unmixing. IEEE Signal Process. Mag. 19, 44–57 (2002).

[b6] Candès E. J. & Wakin M. B. An introduction to compressive sampling. IEEE Signal Process. Mag. 25, 21–30 (2008).

[b7] Duarte M. F. . Single-pixel imaging via compressive sampling. IEEE Signal Process. Mag. 25, 83 (2008).

[b8] Wagadarikar A. A., Pitsianis N. P., Sun X. & Brady D. J. Video rate spectral imaging using a coded aperture snapshot spectral imager. Opt. Express 17, 6368–88 (2009). 19365462 10.1364/oe.17.006368

[b9] Arguello H. & Arce G. R. Code aperture optimization for spectrally agile compressive imaging. J. Opt. Soc. Am. A. 28, 2400–2413 (2011). 10.1364/JOSAA.28.002400 22048307

[b10] Arce G. R., Brady D. J., Carin L. & Arguello H. Compressive coded aperture spectral imaging: an introduction. IEEE Signal Process. Mag. 31, 105–115 (2014).

[b11] Tsai T., Llull P., Yuan X., Carin L. & Brady D. J. Spectral-temporal compressive imaging. Opt. Lett. 40, 4054–4057 (2015). 26368710 10.1364/OL.40.004054

[b12] Liu Z. . Spectral camera based on ghost imaging via sparsity constraints. Sci. Reports 6, 25718 (2016). 10.1038/srep25718 PMC4867594 27180619

[b13] Magalhães F., Abolbashari M., Araùjo F. M., Correia M. V. & Farahi F. High-resolution hyperspectral single-pixel imaging system based on compressive sensing. Opt. Eng. 51, 071406–1 (2012).

[b14] Soldevila F., Irles E., Durán V., Clemente P., Fernandez M., Tajahuerce E. & Lancis J. Single-pixel polarimetric imaging spectrometer by compressive sensing. Appl. Phys. B 113, 551–558 (2013).

[b15] Welsh S. S. . Fast full-color computational imaging with single-pixel detectors. Opt. Express 21, 23068–23074 (2013). 24104222 10.1364/OE.21.023068

[b16] Wang Y., Suo J., Fan J. & Dai Q. Hyperspectral computational ghost imaging via temporal multiplexing. IEEE Photonics Technol. Lett. 28, 288–291 (2016).

[b17] Bian L. . Multispectral imaging using a single bucket detector. Sci. Reports 6, 24752 (2016). 10.1038/srep24752 PMC4840436 27103168

[b18] Schechner Y. Y., Nayar S. K. & Belhumeur P. N. Multiplexing for optimal lighting. IEEE T. Pattern Anal. 29, 1339–54 (2007). 10.1109/TPAMI.2007.1151 17568139

[b19] Edgar M. P. . Simultaneous real-time visible and infrared video with single-pixel detectors. Sci. Reports 5 (2015). 10.1038/srep10669 PMC4650679 26001092

[b20] Watts C. M. . Terahertz compressive imaging with metamaterial spatial light modulators. Nat. Photonics 8, 605–609 (2014).

[b21] Harvey A. R. . Spectral imaging in a snapshot. Prog. Biomed. Opt. Imag. 5694, 110–119 (2005).

[b22] Gorman A. Fletcher-Holmes D. W. & Harvey A. R. Generalization of the Lyot filter and its application to snapshot spectral imaging. Opt. Express 18, 5602–5608 (2010). 20389576 10.1364/OE.18.005602

[b23] Suo J. . A self-synchronized high speed computational ghost imaging system: a leap towards dynamic capturing. Opt. Laser. Technol. 74, 65–71 (2015).

[b24] August Y., Vachman C., Rivenson Y. & Stern A. Compressive hyperspectral imaging by random separable projections in both the spatial and the spectral domains. Applied Optics 52, 46–54 (2013). 10.1364/AO.52.000D46 23545982

[b25] Gehm M. E., John R., Brady D. J., Willett R. M. & Schulz T. J. Single-shot compressive spectral imaging with a dual-disperser architecture. Optics Express 15, 14013–27 (2007). 19550674 10.1364/oe.15.014013

[b26] Li Z., Suo J., Hu X. & Dai Q. Content-adaptive ghost imaging of dynamic scenes. Opt. Express 24, 7328–7336 (2016). 27137022 10.1364/OE.24.007328

[b27] Zhang Z., Ma X. & Zhong J. Single-pixel imaging by means of fourier spectrum acquisition. Nature Communications 6 (2015). 10.1038/ncomms7225 25649009

[b28] Sun M., Edgar M. P., David B. P., Graham M. G. & Miles J. P. Improving the signal-to-noise ratio of single-pixel imaging using digital microscanning. Opt. Express 24, 10476–10485 (2016). 27409871 10.1364/OE.24.010476

[b29] YangA. Y., ZhouZ., BalasubramanianA. G. & SastryS. S. Fast 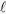 1-minimization algorithms for robust face recognition. Comput. Sc. 22, 3234–3246 (2010).10.1109/TIP.2013.226229223674456

